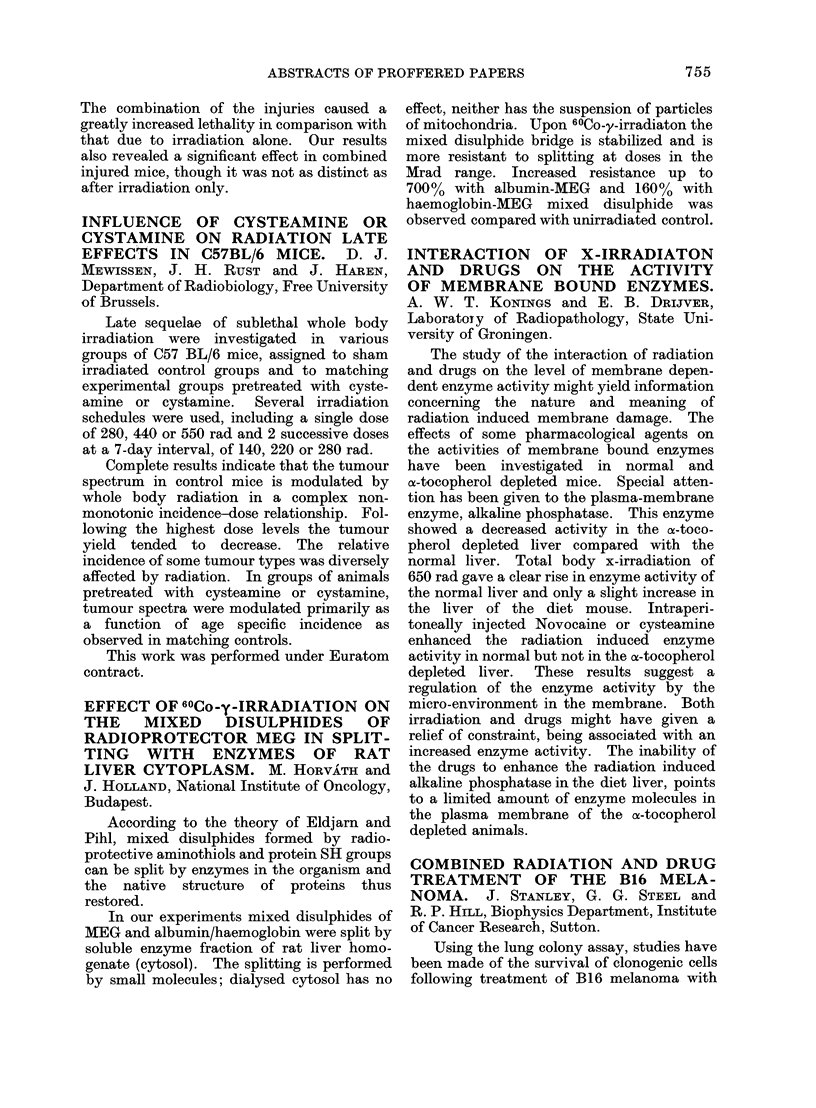# Proceedings: Influence of cysteamine or cystamine on radiation late effects in C57BL/6 mice.

**DOI:** 10.1038/bjc.1975.300

**Published:** 1975-12

**Authors:** D. J. Mewissen, J. H. Rust, J. Haren


**INFLUENCE OF CYSTEAMINE OR CYSTAMINE ON RADIATION LATE EFFECTS IN C57BL/6 MICE.** D. J. MEWISSEN, J. H. RUST and J. HAREN, Department of Radiobiology, Free University of Brussels.

Late sequelae of sublethal whole body irradiation were investigated in various groups of C57 BL/6 mice, assigned to sham irradiated control groups and to matching experimental groups pretreated with cysteamine or cystamine. Several irradiation schedules were used, including a single dose of 280, 440 or 550 rad and 2 successive doses at a 7-day interval, of 140, 220 or 280 rad.

Complete results indicate that the tumour spectrum in control mice is modulated by whole body radiation in a complex non-monotonic incidence–dose relationship. Following the highest dose levels the tumour yield tended to decrease. The relative incidence of some tumour types was diversely affected by radiation. In groups of animals pretreated with cysteamine or cystamine, tumour spectra were modulated primarily as a function of age specific incidence as observed in matching controls.

This work was performed under Euratom contract.